# Effect of Concave Stave on Class I Barrel-Stave Flextensional Transducer

**DOI:** 10.3390/mi12101258

**Published:** 2021-10-17

**Authors:** Duo Teng, Xiaoyong Liu, Feng Gao

**Affiliations:** 1 School of Marine Science and Technology, Northwestern Polytechnical University, Xi’an 710072, China; liuxiaoyong_nwpu@hotmail.com; 2 National Key Laboratory for Underwater Information Processing and Control, Xi’an 710072, China; 3 State Key Laboratory of Solidification Processing, NPU-QMUL Joint Research Institute of Advanced Materials and Structure, School of Materials Science and Engineering, Northwestern Polytechnical University, Xi’an 710072, China; gaofeng@nwpu.edu.cn

**Keywords:** Class I barrel-stave flextensional transducer, concave stave, low frequency, small size, finite element method

## Abstract

To meet the requirements of low frequency, high power, small size and light weight, a type of Class I barrel-stave flextensional transducer employing improved concave stave is presented. As the key component of flextensional transducer, concave stave plays an important role in vibrating efficiently to radiate acoustic energy. The structure of concave stave has a great effect on its behavior. In this paper, the main parameters of concave stave are discussed, especially the effect of radius on flextensional transducer. Both concave stave and transducer are analyzed through finite element method, including mechanical transformation behavior of concave stave and performances of flextensional transducer. On the basis of finite element design, five prototypes employing concave staves with different radii are manufactured and measured. The simulations and tests reveal that concave stave can affect performances of flextensional transducer. A larger radius of concave stave will result in a greater amplification of vibration and a lower resonance frequency of transducer. This can be a feasible way to optimize the resonance frequency or source level of flextensional transducer through adjusting the radius of concave stave in a small range. According to the electrical and acoustical tests, our Class I barrel-stave flextensional transducer is capable of being used as underwater low-frequency small-size projector.

## 1. Introduction

Transducers, as indispensible devices in underwater applications, play an important role [1]. This results from the fact that underwater acoustic wave is comparatively effective way to propagate farther in water [2]. Such transducers are used to convert electrical energy to acoustical energy or vice versa [3]. Their performances have a decisive impact on implementation of underwater application. In order to meet the increasingly demanding requirements for underwater detection and communication, transducers with performances of low frequency, high power, light weight and small size are desired [4]. After several decades of development, some types of low-frequency transducers have been presented, for instance, flextensional transducers [5], free-flooded ring transducers [6], flexural transducers [7], etc. They can all be used as projectors because of their amazing performances of low frequency and high power [8]. Especially, flextensional transducers with compact configurations have gotten more popularity. As excellent underwater projector, flextensional transducer can generally achieve transmitting voltage response of more than 125 dB and source level of more than 190 dB. In essence, when flextensional transducers are electrically excited, complex vibration modes will be produced, including flexural mode of metal shell and extensional mode of drive stack [9]. The term “flextensional” by combining “flexural” and “extensional” indicates the inherence [10]. The first flextensional transducer has been attributed to Hayes. He completed construction and tests for foghorn application in 1929 [11], and later described the theory and design in 1936 [12]. Another well-known inventor mentioned in some literatures is Toulis. He presented an oval shell flextensional transducer for underwater applications in 1950s, and was awarded patents in 1966 [13]. In the later research, for convenience of discussion, flextensional transducers have been classified into Class I–VI by Brigham, Royster and Jones [14,15]. Each Class employed special shell. A Class I flextensional transducer employing a slotted concave shell was described by Somayajula, et al. [16]. Royster presented a Class II flextensional transducer with a shell composed of several individual convex beam segments [17]. The shell of Class III flextensional transducer typically looks like a combination of two Class I shells. A graft-shaped shell, including two slotted convex segments and one ring segment, was studied by Chai et al. for Class III flextensional transducer [18]. A shell in the form of elliptical cylinder was used for Class IV flextensional transducer by Zhou, et al. [19]. Zhang et al. introduced a Class V flextensional transducer with a shell of cymbal caps [20], and Sun et al. researched another cymbal flextensional transducer with concave cymbal caps [21]. Class VII transducer is also known as dogbone transducer, because of dogbone-shaped shell. Moosad et al. investigated the effect of such a shell on Class VII transducer [22]. Except these, more complex shells have also been studied. Bonin and Purcell presented a novel folded shell for flextensional transducer [23]. All the shells mentioned in above flextensional transducers can present differential flexure. If these shells are designed improperly, the results of performance reduction and limitations will be caused to transducer, for example, small source level, inaccurate resonance frequency, etc.

This paper will mainly relate to Class I flextensional transducer and its shell. Class I configuration wins great popularity because of its performances of low frequency, high power, light weight and small size. Appearance perspective, its shell can be concave or convex barrel-shaped. Such a barrel shell can be formed from either a solid-shell or a slotted-shell [24], but more generally be formed by assembling a number of staves [25]. This is so-called Class I barrel-stave flextensional transducer (BSFT). Because the shell, or the staves, are very important for flextensional transducers, analysis of their behavior is significative. In this paper, concave staves used in Class I BSFT will be discussed, including configuration, material, flexural vibration and effects on the performances of transducers.

## 2. Class I Concave Barrel-Stave Flextensional Transducer

**Figure 1:**
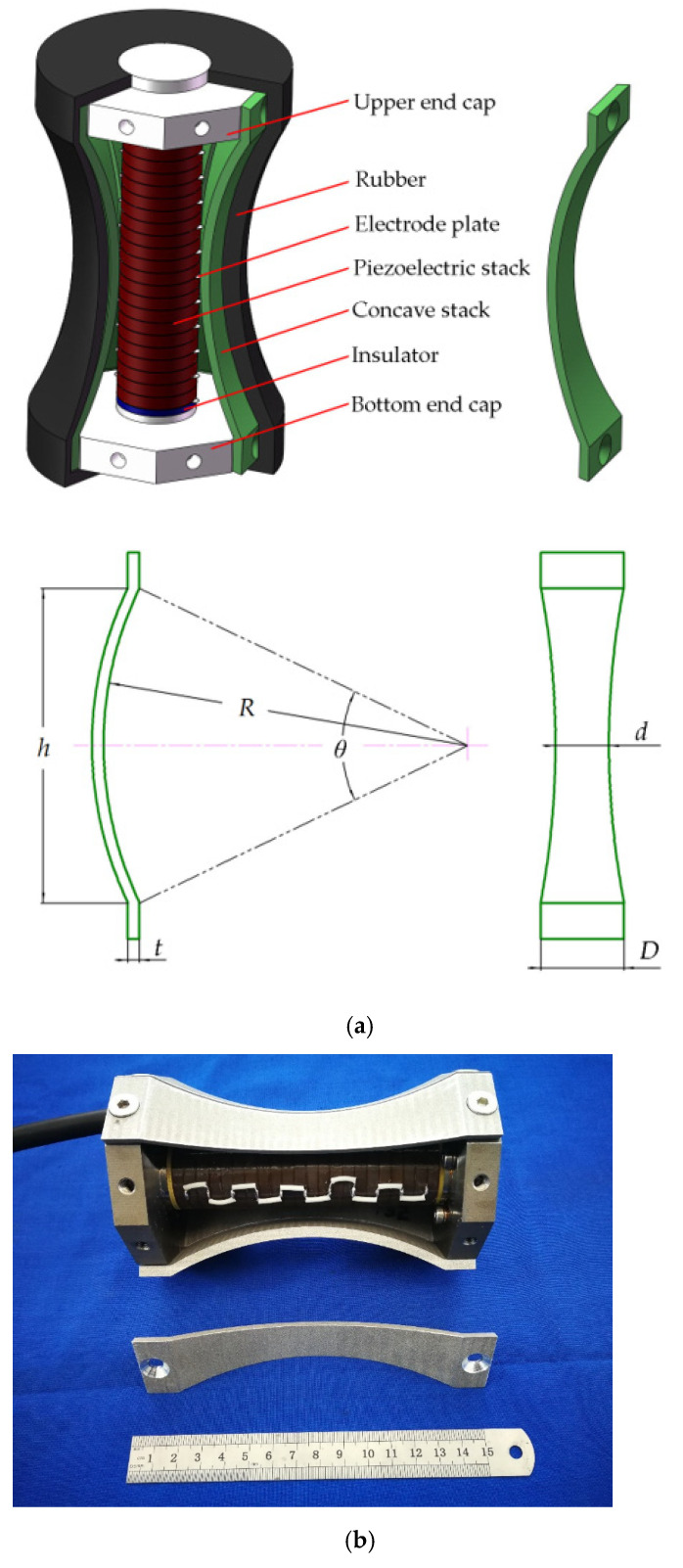
Class I concave barrel-stave flextensional transducer. (**a**) Sketch of Class I barrel-stave flextensional transducer (BSFT) and its concave stave. (**b**) Photograph of Class I BSFT without rubber encapsulation.

Generally, the components of Class I BSFT include drive stack, end caps, prestressed bolt, metal shell, and the other appurtenances. Drive stack is usually piezoelectric ceramic or magnetostrictive rod. In our design, the driver is formed by 22 pieces of PZT4 piezoelectric rings. All the piezoelectric rings are polarized along thickness direction, glued closely together in series and wired electrically in parallel. Piezoelectric stack is fastened to two end caps by prestressed bolt. Each end cap is octagonal, and its edges are matched in assembling the staves. So, there are 8 staves to be employed to form a surrounding shell. A slot with an appropriate width should be kept between adjacent staves. All the slots will play a significant role in resonance frequency and flexural vibrations [26]. Figure 1 illustrates our configuration of BSFT. The adopted staves are concave rather than convex in our design. This is because that a concave shell will present in-phase vibration of all radiating surface on the whole transducer [27]. All the concave staves can be treated as curved beams. Assuming that the radius is *R*, the thickness is *t*, the height is *h*, the width at the clamped ends is *D*, while the width in the central section is *d*. Obviously, *d* is smaller than *D* because all the concave staves should be surrounded to form a concave barrel shell. As a result, concave staves can be treated as beams with variable cross-section. Different from conventional staves, which are mechanically cut from a concave cylinder with thin shell [28], our concave staves are straight instead of curved shape in width, or circumferential, direction. Such staves are easier to be machined through wire-electrode cutting. Moreover, they are advantageous in flexural vibration because of reducing circumferential stiffness.

As an underwater acoustic projector, Class I BSFT will present helpful vibrations for radiating greater low-frequency acoustic power in water [29]. Under an electric drive, extensional vibration of piezoelectric stack will occur, and then two end caps will stretch eight concave staves to excite flexural vibration. That is to say, flexural vibration of barrel shell is derived from extensional vibration of piezoelectric stack. Furthermore, the correlation of above two vibration modes concerns conversion and amplification of piezoelectric stack output displacement, just acting as a mechanical transformer. The vibrations of all the radiating surfaces are in phase. This will enhance the radiating acoustic field, especially for a small size projector.

## 3. Finite Element Analysis of Class I BSFT

There are several methods to design, analyze or optimize piezoelectric transducers, e.g., equivalent circuit method [30], finite element method (FEM) [31], topology optimization method [32], statistical multiple regression analysis method [33], etc. For a Class I BSFT, it is difficult to develop a perfect method, not only because of the coupling of mechanical-electrical-acoustical multiple physical domain [34], but also because of complex vibration modes, including flexural behavior of concave stave as curved beam with variable cross-section. With development of high-performance computer and professional commercial software, FEM has become the prevailing method to design or simulate piezoelectric transducers. This should owe to its advantages of convenient modeling, rapid solution, accuracy result and intuitive illustration [35]. Flextensional transducers with sophisticated configurations and complex boundary conditions can be modeled without large-scale simplifications.

**Figure 2:**
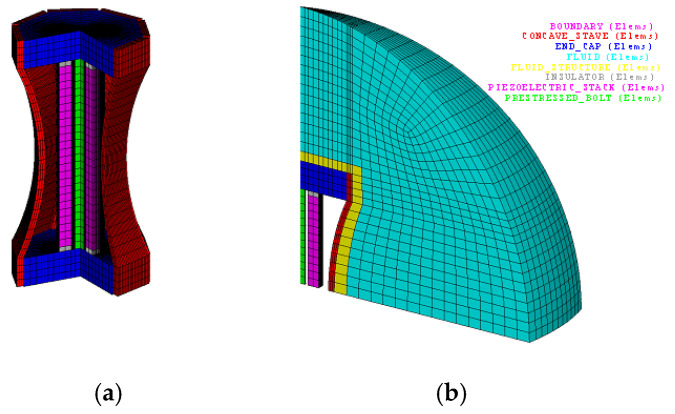
Finite element model of Class I concave barrel-stave flextensional transducer. (**a**) 3/4 model for structure specification; (**b**) 1/32 symmetrical finite element model in water for solution.

In order to discuss the effect of different concave staves on flextensional transducer, a total of 5 Class I BSFTs employing concave staves with different radii have been designed, manufactured and discussed in this paper. The corresponding finite element model is shown in Figure 2. During the process of FEM solution, in order to reduce modeling difficulties and save calculation time, a 1/32 symmetrical finite element model has been built. Some indispensable assumptions have been considered under the premise of little influence on accuracy of solution [36]. It is important to note that the slots between the adjacent staves, with the width of 2 mm, shouldn’t be ignored. The model includes 9464 nodes and 8626 elements. The different colors denote different components of the transducer. The details about sizes and materials are illustrated in Table 1. We will use ANSYS software to solve above model to predict the performances of BSFT. All the following results of FEM are obtained under in-water loading conditions.

**Figure 3:**
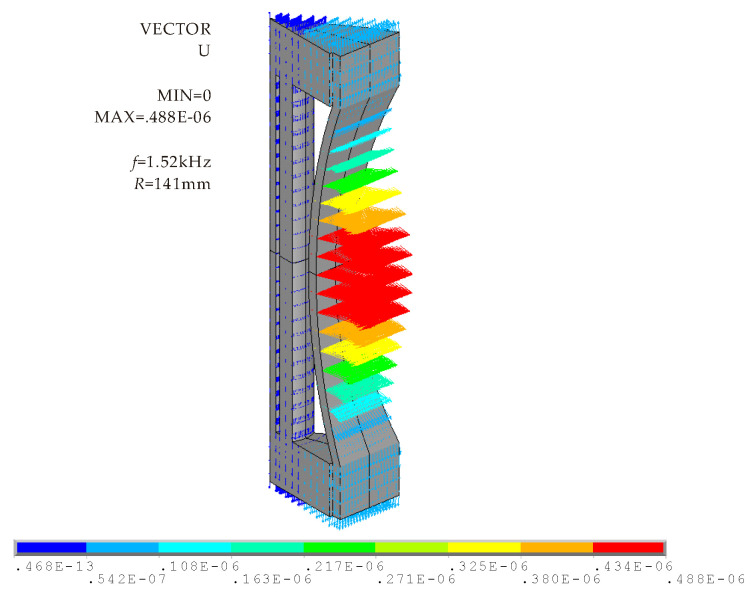
Vector illustration of vibration of Class I BSFT in water (*f* = 1.52 kHz, *R* = 141 mm).

The vibration modes of Class I BSFT will be presented intuitively through FEM. The vector illustration shown in Figure 3 is eighth part of the whole transducer, including one concave stave with radius of 141 mm. The modal frequency is 1.52 kHz, which is the resonance frequency of transducer. The mode shape is complex, but shows regularity. Piezoelectric stack and two end caps present extensional mode, while concave stave presents flexural mode. From a vibration intensity perspective, small longitudinal vibration is transformed to strong bending vibration. The most obvious vibrations occur at intermediate section of concave stave, which is main domain to radiate acoustic energy. Figure 3 indicates that in-phase vibrations are produced on the radiating faces of transducer. That is to say all the radiating faces are vibrating simultaneously outwards or inwards, just with different amplitude. For a small size transducer, such vibrations are helpful for enhancing omnidirectional radiation field.

**Figure 4:**
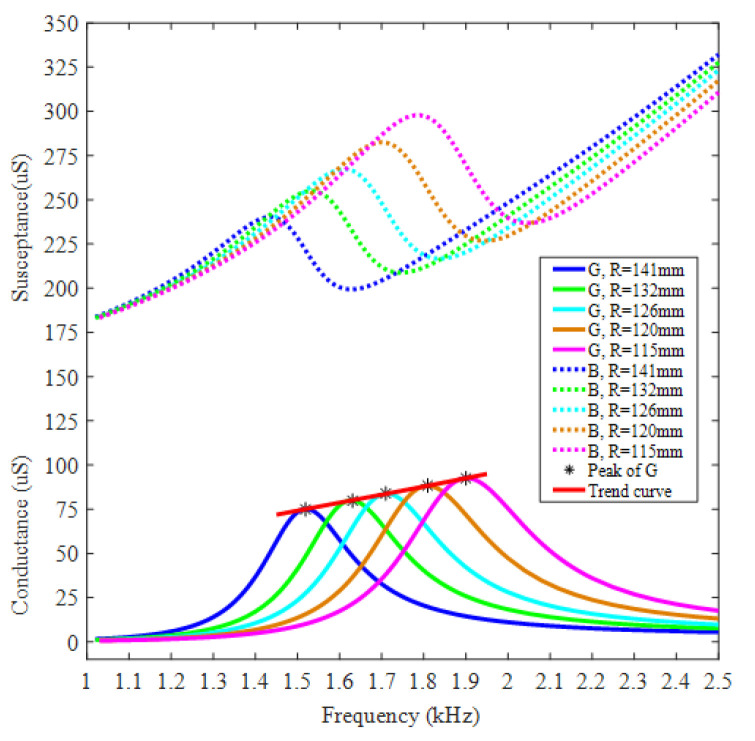
In-water admittance curves of Class I BSFT employing concave staves with different radii.

Figure 4 presents admittance performances of Class I BSFTs with different *R*. An obvious peak appears in each conductance curve. Take for instance the transducer with concave staves of *R* = 141 mm. Resonance characterized by the location of this peak is near 1.52 kHz. This is the frequency of maximum response for transducer driven with a constant voltage. If the acoustic pressure is denoted by *p*(*r*) and the driving voltage of transducer is denoted by *U*, the response in dB for an input of one rms volt is defined as transmitting voltage response in underwater applications, namely

(1) $$\mathrm{TVR}=20\log\left|\frac{p\left(r=1m\right)}{U}\right|+120\;\mathrm{referred}\;\mathrm{to}\;1\;µ\mathrm{Pa}\;\mathrm{at}\;1\;m$$

**Figure 5:**
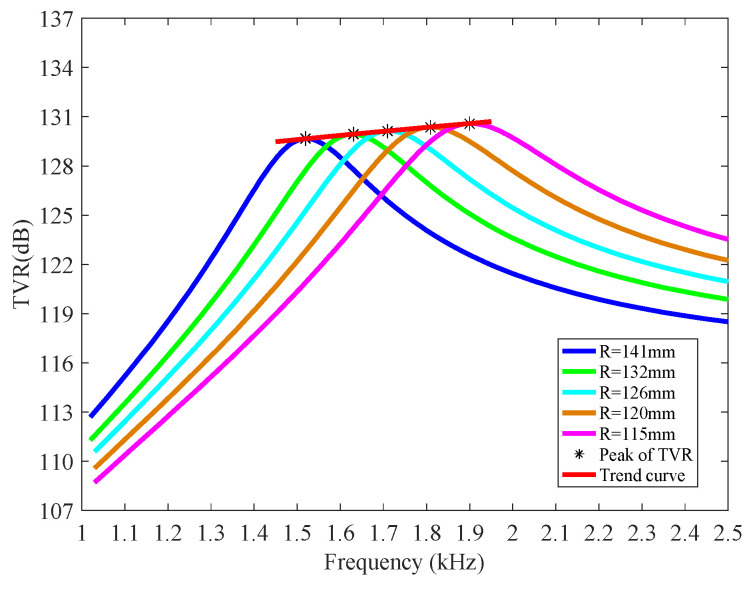
TVR curve of Class I BSFT employing concave staves with different radii.

TVR can reflect the transmitting ability of a projector. The TVR curves shown in Figure 5 indicate the greatest ability of converting electrical energy to acoustical energy when an electroacoustic transducer operates near resonance. The maximum TVR is 129.7 dB at the frequency of 1.52 kHz. Its bandwidth of 3 dB down is about 300 Hz. Therefore, this is a typical narrowband projector.

The other Class I BSFTs, which employ concave staves with different radii of 115,120, 126,132 mm, respectively, have also been analyzed by FEM and illustrated in Figure 4 and Figure 5. All the simulations indicate similar behaviors of transducers, but also present the effect of concave stave on the performances of transducer. The concave stave is the key for BSFTs.

## 4. Concave Stave

For Class I BSFTs, their configurations are well defined. Each part is employed to play the relevant role. Piezoelectric stack produces original vibration. In order to obtain strong longitudinal response from electrical excitation, it is generally formed by 33-mode piezoelectric rings. By contrast, the shell, or the staves, produce key vibration to radiate acoustic energy. Their structures are very important to transducers. The relevant optimizations are also the main point for researchers to improve flextensional transducer.

**Figure 6:**
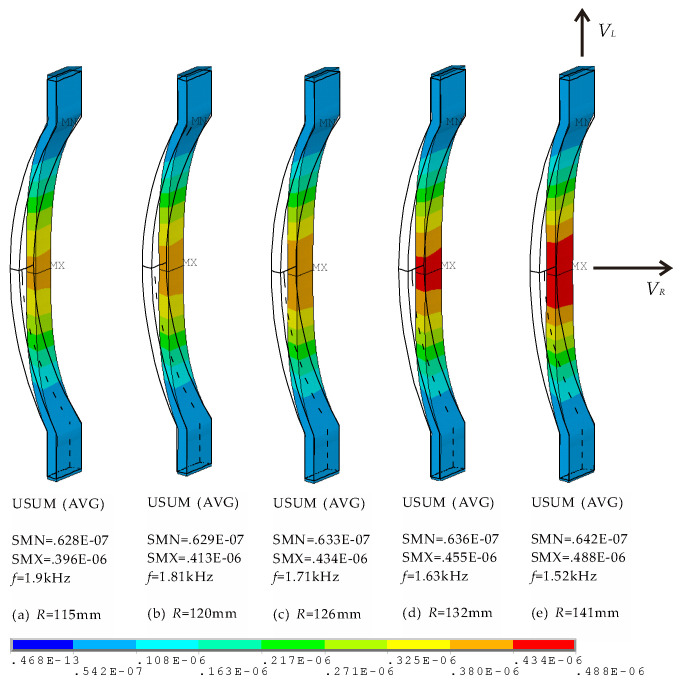
Contour plots of vibration displacements of concave staves with different radii (in water).

In this paper, a special form of concave stave shown in Figure 1 are employed. These staves, as specific implementor of mechanical transformer, are the key to realize conversion of vibrational direction and amplification of output displacement. Especially radius is one of the crucial affecting factors. Figure 6 illustrates vibration displacements (USUM) of different concave staves. Each contour plot corresponds to respective resonance frequency, *f*, which vary with radii of 115, 120, 126, 132, 141 mm. All the concave staves produce desired conversion, but different amplifications. If uniform contour values are specified, Figure 6 reveals that concave staves with larger radius produce the stronger vibration at their intermediate section.

**Figure 7:**
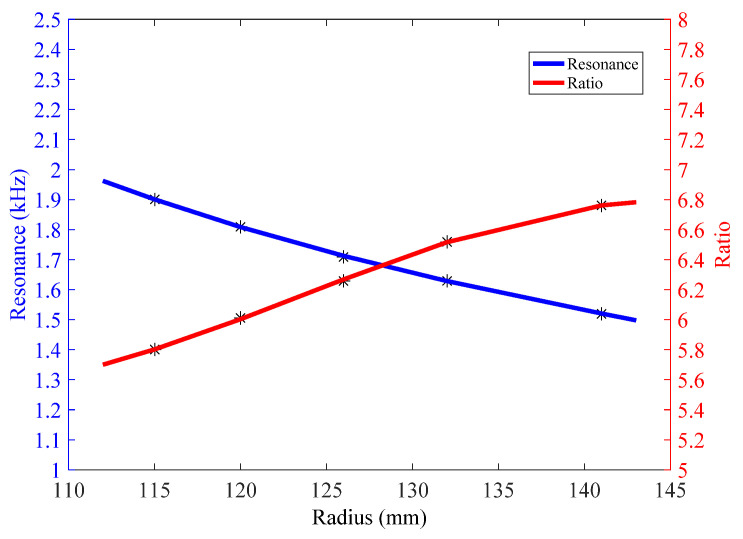
Resonance of transducer and amplifications of vibration, *A,* vs. radius of concave stave (in water).

If average longitudinal vibration of their end planes is designated as *V_L_*, while average bending vibration of their intermediate point is designated as *V_R_*, the ratio, *A* = *V_R_*/*V_L_*, is amplifications of mechanical transformer. Figure 7 reveals that different radii of concave staves produce different amplifications. The ratio is 5.80 when concave stave has the radius of 115 mm, while ratio is 6.76 when *R* = 141 mm. More simulations about concave staves with different radii disclose that the smaller the radius is, the lower the ratio is.

The radii of concave staves also affect performances of transducer. Figure 7 illustrates the change of transducers’ resonances in a limited radius range of concave stave. With enlarging the radius, resonance of corresponding Class I BSFT will be lowered. Finite element simulations reveal that resonances will be changed from 1.9 kHz to 1.52 kHz when the radii of concave staves are set from 115 mm to 141 mm. This is a feasible way to obtain a desired resonance of BSFT just through adjusting the radius of concave stave, while without changing the other components.

Of course, the adjustment of radius will also cause the fluctuations of the other parameters of transducer, such as admittance and TVR. Figure 4 illustrates conductance change of BSFTs with different radii of concave staves. The maximum conductance is largen by 27.2% when radius is lowered from 141 mm to 115 mm. But TVR of BSFT, shown in Figure 5, is not changed obviously. The values stay at level of about 130 dB. There are several factors to influence TVR. If a transducer with any shape has surface velocities in phase at frequencies where the transducer dimensions are small compared to wavelength, the far-field pressure, *p*(*r*), is given approximately [38]

(2) $$p\left(r\right)=j\frac{\rho c}{2r}\frac{\iint_{S}udS}{\lambda }\cdot e^{-jkr}$$

where *u* is normal velocity of radiating surface of transducer, *S* is radiating surface of transducer, *ρ* and *c* are density and sound velocity of water, *λ* is wavelength. The far-field pressure at a distance of *r* can be extrapolated back to the pressure at 1 m by

(3) p(r=1m)=r⋅p(r)

According to Equation (1), when BSFTs are driven by same input voltage, *U*, far-field pressure can reflect consistent change as TVR of transducer. A qualitative comparison between *R* = 115 and *R* = 141, based on *p*(*r*), will reveal the reason of TVR with a little change. When *R* = 141, the resonance is relatively low, the wavelength is relatively long, but the velocity of radiating surface, *u*, is relatively strengthened (shown in Figure 6). Based on Equation (2), a large fluctuation of the far-field pressure will be not caused. In actual engineering development of BSFT, the optimization about radii of concave staves in a small range is useful and significative.

The thickness of concave stave, *t*, will also cause the change of performances of BSFT. The finite element simulations indicate that the thinner the thickness is, the lower the resonance is and the stronger the vibration of concave stave, *V_R_*, is. As the same qualitative reasoning based on Equation (2), the thickness, *t*, will result in a little change in TVR. But thinning the thickness of concave stave will cause reduction of mechanical strength. The risk of breaking concave staves caused by hydrostatic pressure shouldn’t be neglected, especially for deep-submergence BSFTs.

Another contrastive analysis is about the influence of material. If material of concave staves is titanium alloy stead of aluminium alloy and the size including *R* = 141 mm, *t* = 4.5 mm are unchanged, resonance frequency of transducer will be up from 1.52 kHz to 1.56 kHz. If the material is replaced by steel, resonance frequency will be up to 1.6 kHz. The finite element simulations indicate that the material in order of aluminium alloy, titanium alloy and steel will raise resonance frequency, strengthen the vibration, *V_R_*, and improve TVR. Moreover, an obvious increasement of BSFT’s weight is caused. All these parameters, therefore, should be considered to achieve desired performances in design of BSFT.

## 5. Test and Discussion

**Figure 8:**
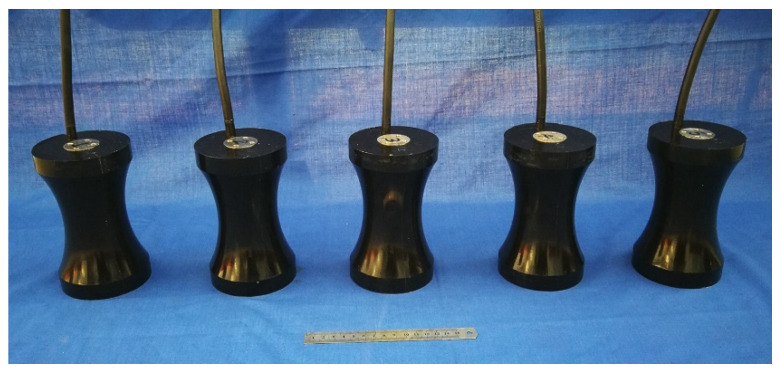
Prototypes of Class I BSFTs with different radii of concave staves.

According to our finite element design, five prototypes of Class I BSFTs have been manufactured and tested. They are all in the same configuration, but employ different concave staves with radii of 115, 120, 126, 132, 141 mm respectively. All the prototypes are encapsulated by polyurethane rubber to prevent water ingression. The used rubber is acoustically transparent because it has the same acoustic characteristic impedance, *ρc*, to water. Figure 8 shows these prototypes. Each one has the diameter of 96 mm, the height of 160 mm and the weight of about 2.27 kg.

**Figure 9:**
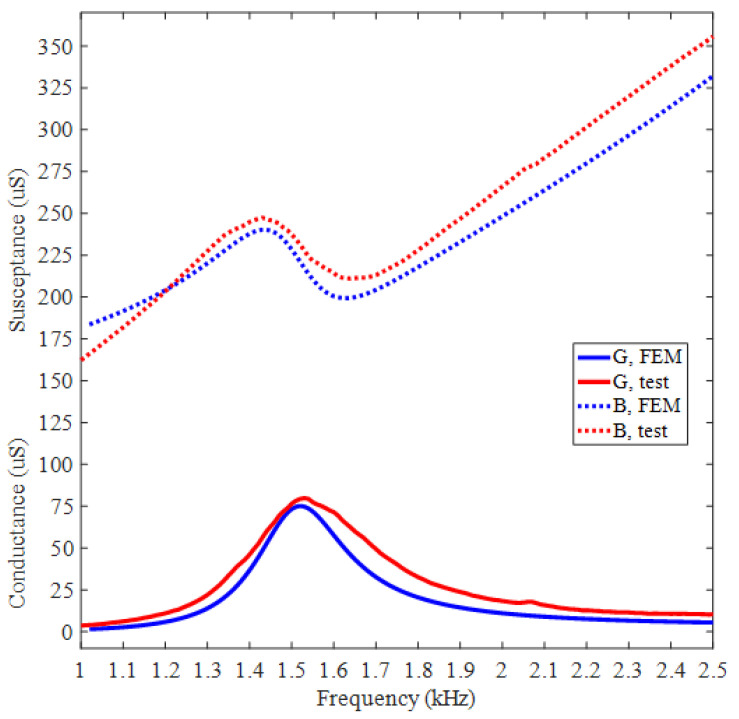
In-water admittance curves of Class I BSFT when *R* = 141 mm (obtained from Agilent 4294A).

**Figure 10:**
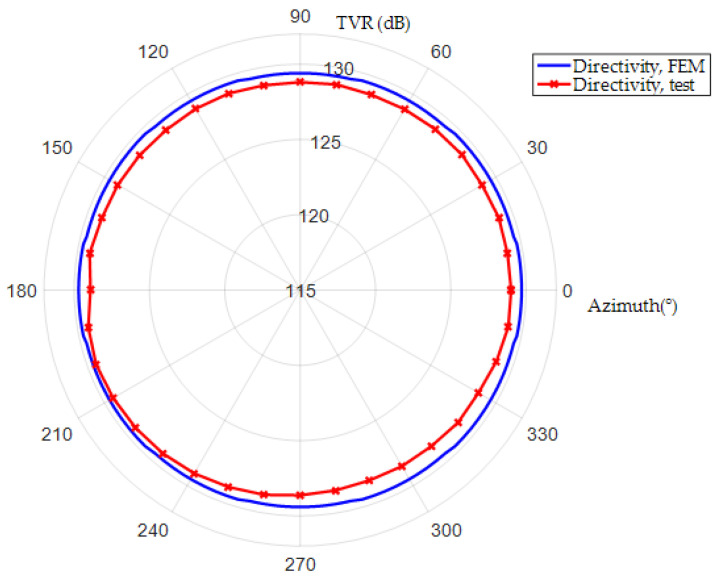
Directivity curves of Class I BSFT in water when *R* = 141 mm, *f* = 1.52 kHz.

**Figure 11:**
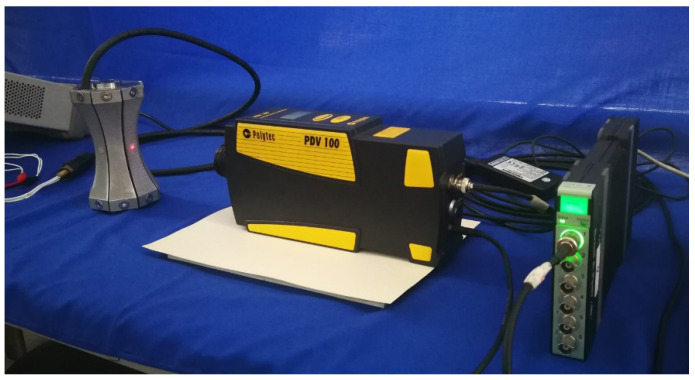
Implementation of vibration test using optical measurement equipment PDV 100 (in air).

The admittances of BSFTs are obtained from precision impedance analyzer Agilent 4294A (Agilent, Santa Clara, CA, USA). Figure 9 shows conductance and susceptance curves of transducer whose concave staves have the radius of 141 mm. The results obtained from FEM are also attached. The peak of tested conductance indicates resonance frequency of 1.53 kHz, which is agreeable with 1.52 kHz of FEM. Figure 10 shows directivities based on TVR of BSFTs in vertical plane. The fluctuation of TVR in different azimuth is within 0.3 dB for FEM, while it is less than 0.96 dB for test. The results reveal that BSFTs are omnidirectional. The other acoustical and vibrational performances have also been tested, including the amplification of vibration, *A*. Figure 11 is implementation of vibration test using optical measurement equipment PDV 100 (Polytec, Waldbronn, Germany). Table 2 illustrates main performances of Class I BSFTs in the case of different radii of concave staves. Resonances of transducers are different from 1.53 kHz to 1.96 kHz, depending on different radius. The tests indicate that resonance of transducer can be adjusted through optimizing the radii of concave staves. The max TVR is obtained at the frequency of resonance for each transducer. All the transducers produce TVR level in accordance with the results of FEM. The maximum deviation is about 1.3 dB for No. 4 transducer. The reason maybe lies in the fabrication process. The amplifications of vibration, *A*, are measured in air. The values are higher slightly than the results of FEM. Maybe the acoustic media or the rubber encapsulating around transducer should be considered. On the whole, the comparisons between FEM and test show the consistency.

As underwater projectors, the transmitting performance of high power is one of important indices. The source level (SL) is capable of producing far-field acoustic pressure on its maximum response axis. Considering the small radiating surface and strong vibration intensity, our BSFTs should be put in depth over 30 m to avoid cavitation when they are transmitting high acoustic power. When satisfying this condition, the driving voltage supplied from power amplifier can be over 1800 V_rms_, and SL of our BSFTs can exceed over 194 dB.

## 6. Conclusions

A type of Class I BSFT with improved concave staves is presented. The performances of transducer and behavior of concave staves are predicted through FEM. The effects of concave staves on BSFTs are studied. Five prototypes employing concave staves with different radii are manufactured. The relevant tests show agreements with the results of FEM. In summary, the following conclusions can be drawn.

(1) As underwater projectors, our Class I BSFTs exhibit the outstanding electrical and acoustical performances. Their advantages of low frequency, high power, small size and light weight are obvious. Especially, their weight of 2.27 kg and size of Φ 96 × 160 mm are inaccessible targets for another classical low-frequency projectors such as free-flooded rings with same resonance frequency. The portability of Class I BSFTs is attractive in their application.
(2) Shell is important for a flextensional transducer to lower its resonance and produce useful vibration. In our configuration, the shell is composed of several improved concave staves, which are curved along height direction while are planar along width direction. This type of concave staves is easy to be machined. Moreover, they present beneficial performances for transducer. Concave stave is the key for our BSFTs.
(3) The radius of concave stave is one of the crucial affecting factors to performances of transducer. The resonance frequency of transducer will be gradually lowered with enlarging the radius of concave stave. In a small range, this is a feasible way to obtained desired resonance of BSFT.
(4) Concave stave is specific implementor for transducer to convert vibration direction and amplify out displacement. The radius of concave stave affects amplification of vibration. A larger radius will result in a greater amplification. But in a small radius range, source level will not be caused in synchronized raising because of the falling of resonance.

## Figures and Tables

**Table 1:** Size and material properties of Class I concave barrel-stave flextensional transducer.

Components	Size	Material	Quantity	Material Property [37]
Piezoelectric stack	22 pieces of piezoelectric rings (Φ 30 × 5 mm with a hole of Φ 13 mm), polarization along thickness direction	PZT-4 piezoelectric ceramic	Density (kg/m^3^)	7600
			Stiffness coefficients matrix (×10^10^ N/m^2^)	$c^{E}=\left[\begin{array}{llllll} 13.9 & 7.78 & 7.43 & 0 & 0 & 0\\ 7.78 & 13.9 & 7.43 & 0 & 0 & 0\\ 7.43 & 7.43 & 11.5 & 0 & 0 & 0\\ 0 & 0 & 0 & 3.06 & 0 & 0\\ 0 & 0 & 0 & 0 & 2.56 & 0\\ 0 & 0 & 0 & 0 & 0 & 2.56 \end{array}\right]$
			Piezoelectric stress matrix (C/m^2^)	$e=\left[\begin{array}{lll} 0 & 0 & -5.2\\ 0 & 0 & -5.2\\ 0 & 0 & 15.1\\ 0 & 0 & 0\\ 0 & 12.7 & 0\\ 12.7 & 0 & 0 \end{array}\right]$
			Relative permittivity matrix	$\frac{\varepsilon^{S}}{\varepsilon_{0}}=\left[\begin{array}{lll} 730 & 0 & 0\\ 0 & 730 & 0\\ 0 & 0 & 635 \end{array}\right]$
Insulator	2 pieces, (Φ 30 × 3 mm with a hole of Φ 13 mm)	Rigid laminated sheet Epoxy resin	Density (kg/m^3^)	1800
			Young’s modulus (N/m^2^)	2.4 × 1010
			Poisson’s ratio	0.38
End cap	2 pieces, Octagon, Inscribed circle diameter of 74 mm, Thickness of 18 mm	Steel	Density (kg/m^3^)	7840
			Young’s modulus (N/m^2^)	2.16 × 1011
			Poisson’s ratio	0.28
Prestressed bolt	M8 × 123.5 mm	Steel		
Concave stave	8 pieces, *R* = 141 mm, *t* = 4.5 mm, *h* = 122 mm, *D* = 28.5 mm, *d* = 15.5 mm	Aluminium alloy	Density (kg/m^3^)	2700
			Young’s modulus (N/m^2^)	7.1 × 1010
			Poisson’s ratio	0.33
Acoustic medium around BSFT		Water	Density (kg/m^3^)	1000
			Sound velocity (m/s)	1481

**Table 2:** Main performances of Class I BSFTs (comparison between FEM and test).

No.	Radius (mm)	Resonance (kHz) FEM Test		Max TVR (dB) FEM Test		Amplification FEM Test	
1	115	1.9	1.96	130.6	129.6	5.80	5.98
2	120	1.81	1.85	130.4	130.1	6.01	6.40
3	126	1.71	1.71	130.1	129.3	6.26	6.33
4	132	1.63	1.64	129.9	128.6	6.52	6.57
5	141	1.52	1.53	129.7	129.0	6.76	6.95
